# Acupuncture for the sequelae of Bell’s palsy: a randomized controlled trial

**DOI:** 10.1186/s13063-015-0777-z

**Published:** 2015-06-03

**Authors:** Hyo-Jung Kwon, Jun-Yong Choi, Myeong Soo Lee, Yong-Suk Kim, Byung-Cheul Shin, Jong-In Kim

**Affiliations:** Facial Palsy Center, Department of Acupuncture & Moxibustion, College of Korean Medicine, Kyung Hee University, Seoul, Republic of Korea; National Clinical Research Center for Korean Medicine, School of Korean Medicine, Pusan National University, Yangsan, Republic of Korea; Clinical Research Division, Korea Institute of Oriental Medicine, Daejeon, Republic of Korea

**Keywords:** Acupuncture, Randomized controlled trial, Sequelae of Bell’s palsy

## Abstract

**Background:**

Incomplete recovery from facial palsy results in social and physical disabilities, and the medical options for the sequelae of Bell’s palsy are limited. Acupuncture is widely used for Bell’s palsy patients in East Asia, but its efficacy is unclear.

**Methods:**

We performed a randomized controlled trial including participants with the sequelae of Bell’s palsy with the following two parallel arms: an acupuncture group (*n* = 26) and a waiting list group (*n* = 13). The acupuncture group received acupuncture treatments for 8 weeks, whereas the waiting list group did not receive acupuncture treatments during the 8-week period after randomization. The primary outcome measure was change in the Facial Disability Index (FDI) social and well-being subscale at week 8. We also analyzed changes in the FDI physical function subscale, the House–Brackmann score, the Sunnybrook Facial Nerve Grading system, lip mobility and stiffness at 5 and 8 weeks after randomization. An intention-to-treat analysis was applied.

**Results:**

The acupuncture group exhibited greater improvements in the FDI social score (mean difference, 23.54; 95 % confidence interval, 12.99 to 34.08) and better results on the FDI physical function subscale (mean difference, 21.54; 95 % confidence interval, 7.62 to 35.46), Sunnybrook Facial Nerve Grading score (mean difference, 14.77; 95 % confidence interval, 5.05 to 24.49), and stiffness scale (mean difference, −1.58; 95 % confidence interval,−2.26 to −0.89) compared with the waiting list group after 8 weeks. No severe adverse event occurred in either group.

**Conclusion:**

Compared with the waiting list group, acupuncture had better therapeutic effects on the social and physical aspects of sequelae of Bell’s palsy.

**Trial registration:**

Current Controlled Trials ISRCTN43104115.

**Electronic supplementary material:**

The online version of this article (doi:10.1186/s13063-015-0777-z) contains supplementary material, which is available to authorized users.

## Background

Bell’s palsy is a sudden idiopathic paralysis of one side of the face that typically recovers within 6 months [1]. The condition occurs in 30/100,000 individuals per year, and approximately 30 % of patients have sequelae, such as unrecovered paresis, contracture of facial muscles, facial spasms, or synkinesis [2]. Because most symptoms recover within 6 months, the sequelae of Bell’s palsy are defined as facial palsy conditions that persist 6 months after the onset of symptoms [3].

Continuing asymmetry of the facial appearance and impaired function from the sequelae of Bell’s palsy can affect the functions of drinking, eating, and speaking. In addition, because facial symmetry frequently determines an individual’s appearance or influences interpersonal attraction [4], continuing asymmetry can affect psychological and social behaviors, thereby aggravating personal quality of life [5].

Most major treatments for Bell’s palsy, such as the administration of steroid and antiviral agents, are focused on the acute state of the disease [6, 7]. Steroids are clinically effective when initiated within 72 hours of the onset of Bell’s palsy, whereas clinical evidence for antiviral monotherapy for acute Bell’s palsy is not available [6, 8, 9]. Among invasive treatments, there is no consensus favoring one intervention over another, such as the injection of botulinum toxin A or surgical reconstruction and physiotherapy [4, 10–12]. No treatment has been demonstrated to be effective in the sequela stage of Bell’s palsy except mime therapy, the clinical effectiveness of which has been demonstrated in a randomized controlled trial [13].

Acupuncture is a safe treatment for a variety of symptoms, including Bell’s palsy [14, 15]. Whether alone or with drug therapy, favorable effects of acupuncture on the disease response rate in the acute stage of Bell’s palsy have been observed. The lack of conclusive appraisals of the effects of acupuncture on Bell’s palsy is attributable to the limitations of clinical studies and errors in study design [16–18]. A large-scale clinical trial that investigated the effects of acupuncture on Bell’s palsy was recently conducted [19]. The findings of this study indicated that manipulated acupuncture could be advantageous compared with non-manipulated acupuncture; however, the study focused on the acute stage of Bell’s palsy. There is no randomized controlled trial evidence regarding the efficacy of acupuncture for sequelae of Bell’s palsy. In this context, we conducted a randomized study of acupuncture for patients affected by sequelae of Bell’s palsy, and we report that its safety and efficacy adhere to the Consolidating Standards of Reporting Trials (CONSORT) statement (see Additional file 1) [20, 21] and the Standards for Reporting Interventions in Controlled Trials of Acupuncture [22].

## Methods

The protocol for this study was published in detail during the trial [23].

### Participants

This study was conducted at Kyung Hee University Hospital of Korean Medicine (Seoul, Korea). The participants were recruited from August 2010 to July 2011, and follow-up was continued until September 2011. Eligible participants with sequelae of Bell’s palsy who signed the informed consent form were recruited.

### Ethics

The study was approved by the institutional ethics review board of Kyung Hee University Hospital of Korean Medicine on June 11, 2010 (approval no. KOMC MIRB 2010–01), and was conducted according to the provisions of the Declaration of Helsinki and Good Clinical Practice guidelines [24]. We obtained written informed consent from every participant.

### Inclusion and exclusion criteria

We included patients who met the following criteria: diagnosed with Bell’s palsy at least six months prior to screening; aged 18–65 years; and scores of less than 70 on the Facial Disability Index (FDI) physical subscale and less than 80 on the FDI social subscale [25]. We excluded participants who had at least one of the following conditions: secondary facial palsy from infection; multiple neuritis; tumors invading the temporal bone; brain contusion or stroke; Ramsay–Hunt syndrome [26]; bilateral or recurrent facial palsy; and serious conditions requiring medical intervention, such as severe hypertension, uncontrolled diabetes, past or current malignant tumors, dyslipidemia, liver or kidney dysfunction, anemia, active pulmonary tuberculosis, hemorrhagic tendency or other infectious or systemic diseases inappropriate for treatment with acupuncture.

### Study procedure

We used the following patient exclusion criteria to avoid confounding effects that could influence the outcome measures: oral administration of steroids or antiviral drugs (acyclovir, valacyclovir, famciclovir, or ganciclovir); a history of surgery for facial palsy, such as facial nerve decompression or reconstruction of the facial nerve or muscle; or a history of acupuncture, moxibustion, vesiculation or massage therapy within the previous three months. We also excluded pregnant or lactating women. Patients whose vital signs were unstable could not participate in the trial and, after randomization, we monitored the vital signs of every participant in both groups at every visit.

A no-acupuncture waiting list group was used as the control because sham acupuncture could not be substituted as a physiologically inert placebo [27].

Over-the-counter drugs for colds, dyspepsia, and headaches were allowed. However, over-the-counter drugs of unknown components or those indicated for Bell’s palsy-related symptoms were not allowed. The participants were instructed to disclose their medication use prior to enrollment. Records of the participants’ drug histories were obtained at each visit, and participants were requested to inform the clinical research team of any changes to their medication or supplement regimens. Additional acupuncture treatments, herbal medicines, or medical interventions elsewhere were not allowed throughout the study.

### Interventions

Acupuncture was performed by specialists in Korean medicine who had graduated from a Korean medical school with a 6-year course and were licensed in Korean Medicine from the Korean Ministry of Health and Welfare. In addition, the specialists were trained in acupuncture and moxibustion medicine in a 4-year residency program. The acupuncture group received acupuncture treatments three times per week for 8 weeks for a total of 24 sessions. A 0.20 mm (diameter) × 30 mm (length) disposable needle (Dongbang Acupuncture, Inc., Boryeong, Korea) was inserted into each acupuncture point to a depth of 5–30 mm; the needle retention time was 10 minutes, depending on the selected points (Table 1), and patients were in the supine position. The treatment required 18 needles at 12 points [28], which were selected on the basis of acupuncture specialist forums and a Korean acupuncture textbook (Table 1) [29]. All participating acupuncture specialists used de-qi sensation techniques to manually manipulate and maintain the needles for a 10-minute period, with manipulation at the start and end of the 10-minute period.

**Table 1:** Acupuncture points and needling procedure

Acupuncture point	Rationale	Direction	Manipulation method	Depth (mm)
ST4 (unaffected side)	Dispel wind and relieve pain indirectly in affected buccal side	Transversely toward ST6	Mild reinforcing-reducing method using clockwise-counterclockwise rotation of needle until de-qi is achieved	20–30
ST6 (unaffected side)	Dispel wind and relieve pain indirectly in affected buccal side	Transversely toward ST4	Mild reinforcing-reducing method using clockwise-counterclockwise rotation of needle until de-qi is achieved	20–30
ST1 (affected side)	Facilitate movement in affected infra-orbital lesion	Transversely toward the eye	Mild reinforcing-reducing method using clockwise-counterclockwise rotation of needle until de-qi is achieved	5–10
EX-HN4 (affected side)	Facilitate movement in affected supra-orbital lesion	Transversely toward the eye	Mild reinforcing-reducing method using clockwise-counterclockwise rotation of needle until de-qi is achieved	5–10
TE23 (affected side)	Facilitate movement in affected supra-orbital lesion	Transversely toward the ear	Mild reinforcing-reducing method using clockwise-counterclockwise rotation of needle until de-qi is achieved	20–30
LI20 (affected side)	Facilitate movement in affected nasolabial lesion	Obliquely along the nasolabial sulcus toward the root of the nose	Mild reinforcing-reducing method using clockwise-counterclockwise rotation of needle until de-qi is achieved	20–30
TE17 (both sides)	Benefit head or face and ears	Perpendicular to skin	Mild reinforcing-reducing method using clockwise-counterclockwise rotation of needle until de-qi is achieved	20–30
ST9 (both sides)	Regulate Qi and blood of whole body	Perpendicular to skin	Mild reinforcing-reducing method using clockwise-counterclockwise rotation of needle until de-qi is achieved	20–30
LI10 (both sides)	Regulate Qi and blood of whole body	Perpendicular to skin	Reinforcing method using clockwise rotation of needle until de-qi is achieved for tonification	20–30
LI4 (both sides)	Regulate face and head area, tonify Qi	Perpendicular to skin	Reinforcing method using clockwise rotation of needle until de-qi is achieved for tonification	20–30
ST36 (both sides)	Tonify Qi and blood of whole body	Perpendicular to skin	Reinforcing method using clockwise rotation of needle until de-qi is achieved for tonification	20–30
GB34 (both sides)	Treat soft tissue anywhere in body; treat contractions, cramp, pain, spasm, weakness, numbness, paralysis	Perpendicular to skin	Reinforcing method using clockwise rotation of needle until de-qi is achieved for tonification	20–30

The participants in the waiting list group were told that they would receive the identical acupuncture treatment for 8 weeks after the randomization.

### Outcomes

The primary outcome measure was the FDI social score after 8 weeks of acupuncture treatment or the waiting list period. The FDI scoring system consists of a physical score and a social score; the reliability and validity of these scores have been established [25]. The participants answered five multiple-choice questions related to problems associated with facial muscle function during the previous month.

The secondary outcome measures included the FDI social score at week 5, the FDI physical score, the House–Brackmann grade [30], lip mobility (lip length and snout indices) [31], and stiffness scales [13] at weeks 5 and 8. The House–Brackmann grade has been tested for reliability [32], and the lip length and snout indices are highly correlated with the House–Brackmann grade [33]. The stiffness scale is a simple five-point scale for facial stiffness (1 = no stiffness; 5 = very stiff) [13].

### Sample size

The sample size was calculated using the SAS statistical package program (version 9.1.3; SAS Institute, Inc., Cary, NC, USA) based on the results of a mime therapy trial that included participants with sequelae of Bell’s palsy; the design of that trial was a randomized parallel waiting list controlled trial [13]. Because no clinical trial has demonstrated efficacy for sequelae of Bell’s palsy except the trial using mime therapy, we assumed that the effect size of our invasive acupuncture therapy would be at least equal to that of non-invasive mime therapy. In addition, the allocation ratio was 2:1, such that fewer participants were allocated to the waiting list group.

In the mime trial [13], the mean difference in the follow-up FDI social score between the groups was 14.5, and the pooled standard deviation was 14.5 within each group. The alpha value and power were 0.05 and 80 %, respectively. From these values, we calculated the sample sizes for independent two-sample *t* tests (two-tailed) as 26 for the acupuncture group and 13 for the waiting list group, allowing for a 20 % dropout rate. This calculation assumed that the mean difference in the FDI social score between the groups was sufficiently important in an expert discussion between two clinicians who have worked in Bell’s palsy clinics for more than 10 years.

### Randomization, allocation concealment, implementation and blinding

Computerized randomization was performed by an outside researcher who was not in direct contact with the participants. Following the baseline assessment, the eligible participants were assigned to two groups. All subjects were informed that there was a possibility that they would be allocated to the waiting list control group (namely, the second type of acupuncture group), and in that case, they would be required to wait 8 weeks prior to receiving acupuncture for 8 weeks. To eliminate detection or observation bias [34], the assessor was blinded to the group allocation. The investigator performing the acupuncture treatment could not be intrinsically blinded; however, the investigator was not allowed to communicate with the participants or the assessor regarding the treatment procedures and outcomes. A sealed envelope that contained the allocation sequence number for each patient was opened after each patient met the eligibility criteria and informed consent was provided. A balanced block randomization was used to assign 26 participants to the acupuncture group and 13 participants to the waiting list group.

### Statistical analysis

The analysis was performed using an intention-to-treat population that comprised all randomized participants, regardless of the actual treatment received. Statistical analysis was performed using the SAS statistical package program (ver. 9.1.3; SAS Institute, Inc., Cary, NC, USA), and the level of significance was established at α = 0.05. We used the last observation carried forward to impute missing data.

All data were analyzed descriptively. Among the primary and secondary outcome measures, the FDI, House–Brackmann grade, and stiffness scale variables were discrete ordinal variables, whereas lip mobility was recorded as a continuous variable. The mean of differences from the baseline values to week 5 or week 8 was compared between the two groups using two-sample *t* tests. For baseline values of the outcome measurements that were significantly different between the groups, an analysis of covariance (ANCOVA) was performed using the imbalanced variables as the covariates and the assigned group as the fixed factor.

## Results

**Fig. 1:**
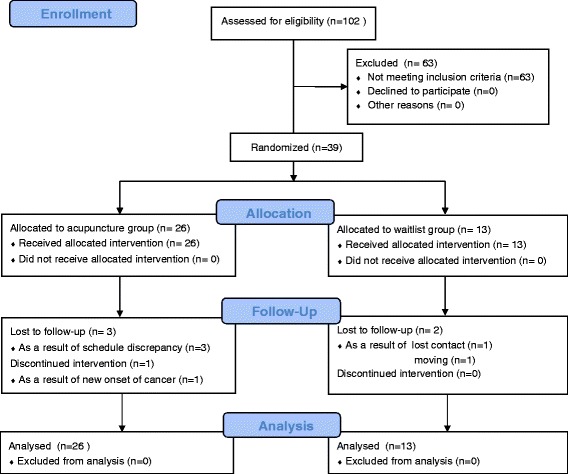
Trial flow

A total of 102 volunteers were screened with the eligibility criteria; 39 participants were randomly allocated to the acupuncture or waiting list groups. Of the 39 initially randomized subjects, 4 participants in the acupuncture group and 2 participants in the waiting list group dropped out during the study period, resulting in an attrition rate of 15 %. For the acupuncture group, the reasons for dropout were schedule discrepancies (*n* = 3) and a new onset of liver cancer (*n* = 1). For the waiting list group, the reasons for dropout were moving (*n* = 1) and loss of contact (*n* = 1). The trial flow is outlined in Fig. 1.

The baseline characteristics of the acupuncture group and the waiting list group were similar; however, the baseline measurements of the lip length and the snout indices for evaluating lip mobility were not similar (Table 2). At the baseline visit, all participants in both groups exhibited stable vital signs, which were maintained throughout the trial. No over-the-counter drug use violations occurred in either group.

**Table 2:** Baseline characteristics of participants

	Acupuncture group (*n* = 26)	Control group (*n* = 13)	*P* ^a^
Sex:			
Male (*n*)	14	4	0.31^b^
Female (*n*)	12	9	
Affected side:			
Left (*n*)	6	7	0.07^b^
Right (*n*)	20	6	
Age (years, mean (SD))	50.85 (10.48)	50.54 (11.48)	0.93
Blood pressure (mmHg):			
Systolic	133.31 (18.31)	124.8 (18.03)	0.18
Diastolic	77.46 (12.87)	79.08 (12.57)	0.71
Duration of facial palsy (months, mean (SD))	94.27 (78.70)	126.15 (119.73)	0.32
Facial Disability Index, social score (mean (SD))	52.77 (16.04)	52.30 (18.36)	0.94
Facial Disability Index, physical score (mean (SD))	46.35 (14.25)	51.15 (14.31)	0.33
Sunnybrook Facial Nerve Grading (mean (SD))	28.19 (21.45)	25.23 (15.30)	0.66
House–Brackmann grade:			
Mild to moderate dysfunction (2–3)	17	7	0.51^b^
Moderately severe to severe dysfunction (4–5)	9	6	
Lip mobility:			
Lip length index (mean (SD))	22.56 (8.62)	17.23 (6.76)	0.06
Snout index (mean (SD))	27.93 (7.94)	21.06 (9.31)	0.02
Facial stiffness^c^ (mean (SD))	4.19 (0.85)	4.00 (0.91)	0.52

SD, standard deviation

^a^Independent two-sample *t* test, unless indicated otherwise

^b^Fisher’s exact test

^c^Numerical rating scale from 1 to 5, where 1 indicates no stiffness and 5 indicates very stiff

### Outcomes

At 8 weeks, the FDI social and physical scores, Sunnybrook Facial Nerve Grading scores, and stiffness index were significantly better in the acupuncture group compared with the waiting list group (Table 3).

**Table 3:** Outcome changes in acupuncture and waiting list groups

		Change from baseline (mean (SD))		Mean difference (95 % confidence interval)
		Acupuncture group, *N* = 26	Control group, *N* = 13	
Facial Disability Index, social score	Week 5	18.92 (18.61)	−0.62 (12.95)	19.54 (7.83, 31.22)
	Week 8	21.69 (21.47)	−1.85(11.10)	23.54 (12.99, 34.08)
Facial Disability Index, physical score	Week 5	23.65 (19.47)	−0.38 (12.98)	24.04 (13.37, 34.71)
	Week 8	26.16 (21.65)	4.62 (16.89)	21.54 (7.62, 35.46)
Sunnybrook Facial Nerve Grading	Week 5	20.92 (16.81)	6.54 (7.26)	14.38 (6.55, 22.21)
	Week 8	24.54 (16.24)	9.77 (8.07)	14.77 (5.05, 24.49)
House–Brackmann grade	Week 5	−0.42 (0.86)	−0.15 (0.99)	−0.27 (−0.88, 0.35)
	Week 8	−0.62 (0.85)	−0.38 (0.96)	−0.23 (−0.93, 0.40)
Lip mobility				
Lip length index	Week 5	0.67 (4.36)	−1.72 (7.41)	3.39 (0.31, 6.48)^a^
	Week 8	1.00 (7.12)	−0.25 (4.45)	2.83 (−1.51, 7.16)^a^
Snout index	Week 5	1.84 (5.94)	1.28 (3.23)	2.15 (−1.49, 5.80)^a^
	Week 8	2.36 (6.81)	−0.80 (4.49)	5.05 (0.76, 9.33)^a^
Facial stiffness^b^	Week 5	−1.35 (1.09)	−0.08 (0.64)	−1.27 (−1.94, −0.60)
	Week 8	−1.58 (1.27)	0.00 (0.82)	−1.58 (−2.26, −0.89)

SD, standard deviation

^a^Adjusted for baseline values because of significant or marginally significant baseline differences

^b^Numerical-rating scale from 1 to 5, where 1 indicates no stiffness and 5 indicates very stiff

The FDI social and physical scores, Sunnybrook Facial Nerve Grading scores, and stiffness index were significantly better in the acupuncture group than in the control waiting list group as early as 5 weeks after the initiation of acupuncture (Table 3).

There was no statistically significant difference in House–Brackmann grade between the groups at week 5 and week 8 (Table 3). In the acupuncture group, six participants with moderately severe to severe facial dysfunction at baseline improved to a mild to moderate level at week 8, and no aggravated cases occurred. This change was significantly different within the groups, according to McNemar’s test. No significant change in the House–Brackmann grade was observed in the control group in the within-group analysis, as determined using McNemar’s test (Table 4).

**Table 4:** House–Brackmann grade change within groups

		Baseline	Week 5	Week 8
Acupuncture group	House–Brackmann grade:			
	Mild to moderate dysfunction (2–3)	17	22	23
	Moderately severe to severe dysfunction (4–5)	9	4 (improved, 5; aggravated, 0)	3 (improved, 6; aggravated, 0)
	*P*		0.07^a^	0.04^a^
Control group	House–Brackmann grade:			
	Mild to moderate dysfunction (2–3)	7	9	9
	Moderately severe to severe dysfunction (4–5)	6	4 (improved, 3; aggravated, 1)	4 (improved, 3; aggravated, 1)
	*P*		0.61	0.61

^a^McNemar’s test between follow-up and baseline test

### Safety

No adverse events were reported apart from minor bleeding and bruising.

## Discussion

In this study, acupuncture had a beneficial effect on sequelae of Bell’s palsy by relieving the social and physical impairments experienced by the participants. In addition, acupuncture improved facial nerve function and stiffness following Bell’s palsy. The effects of acupuncture treatment on strengthening the facial muscles are uncertain.

We identified significant positive improvements in FDI scores. In addition, we identified positive results for the change in the Sunnybrook Facial Nerve Grading score in the following three aspects: facial symmetry, movement asymmetry, and synkinesis. Acupuncture appears to influence facial nerve function positively even at long periods after the onset of Bell’s palsy. There was an obvious decrease in facial stiffness in the acupuncture group.

The focal effect of acupuncture on paralysis could be attributed, in part, to the local effects of acupuncture in stimulating nerve fibers in the skin and muscle [35]. The psychosomatic effects of acupuncture on the autonomic nerve system by regulating Qi probably contributed to the improvement of other outcomes, such as the FDI social score [36].

The change in House–Brackmann grade was not significant between groups. This lack of significance might reflect the nature of the House–Brackmann grade, which is a gross evaluation system that is unable to account for regional differences in the face [30]. However, the improvement of the House–Brackmann grade within the acupuncture group was significant: six of nine patients with moderately severe to severe dysfunction (House–Brackmann grade 4–5) displayed improvement of their House–Brackmann grade score to 2–3, indicating mild to moderate dysfunction (Table 4). Our result is too ambiguous to draw a conclusion regarding the effects of acupuncture in improving lip mobility because there was a significant change in the lip length index at week 5. However, the difference between the groups was not significant at week 8, and the between-group change in the stiffness index was significant only at week 8. This significance was attained by adjusting the baseline variables, and the decrease in the stiffness index in the control group between weeks 5 and 8 might have influenced the difference. Because the onset of Bell’s palsy was diverse in both groups, a sudden impairment of lip mobility is improbable; thus, we can assume that there may have been inaccuracies in the caliper measurement of the lip length by the assessor. Muscles tremble during maximum contraction and relaxation, and it is difficult to hold the maximum moment. The use of computerized video equipment might provide a more accurate measurement and could eliminate bias.

There have been no trials examining sequelae of facial palsy due solely to Bell’s palsy [16]. Therefore, we inevitably established new inclusion criteria for disease duration and FDI scale. In this trial, we focused on whether acupuncture therapy could relieve the symptoms of sequelae of Bell’s palsy. Consequently, the onset of palsy in the participants was important. Among Bell’s palsy cases, 70 % improve before 6 months [1, 2], while 31 % [37] continue to experience sequelae symptoms, such as long-standing paralysis, contractures and synkinesis, which occurs at 23.9–39.0 weeks after the onset of facial palsy [38]. Therefore, we selected patients with Bell’s palsy whose onset was at least 6 months.

For the FDI scale, we selected patients with scores of less than 70 on the FDI physical subscale and less than 80 on the FDI social subscale. These cut-off limits were determined from the result of the mime trial on which we based our sample size [13]. In the mime trial, the FDI physical subscale changed from a mean of 56.8 to a mean of 73.5 in the experimental group and from a mean of 63.2 to a mean of 59.6 in the control group. The FDI social subscale changed from a mean of 68.6 to a mean of 80.7 in the experimental group and from a mean of 72.6 to a mean of 66.2 in the control group. Therefore, we considered cut-off limits of less than 70 on the FDI physical subscale and less than 80 on the FDI social subscale adequate as baseline values to embrace a wide range of grades of sequelae and detect improvement. In addition, we hypothesized that patients with better facial function and FDI scores above the cut-off limits would not gain sufficient improvement from acupuncture therapy for detection in the FDI score. To avoid confounding effects of a past history of steroid or antiviral agent administration, we did not recruit patients who had ever received these drugs.

There are limitations to this study. One limitation is the selection of an appropriate control for an acupuncture clinical trial. Despite our efforts to maintain the expectation of waiting list patients for acupuncture therapy by providing acupuncture treatment after the end of the trial, the “placebo” effect could not be ruled out in the acupuncture group without having a sham acupuncture control group [39, 40]. The complete inertness of a sham acupuncture treatment cannot be guaranteed because any minimal physical contact on the skin might activate the Aβ- and C-fibers [41, 42]; thus, we did not employ a sham acupuncture treatment. In addition, we did not perform electrodiagnostic testing procedures, such as electroneurophysiological testing and facial electromyography, for several reasons. Electrophysiological assessment is used for complete facial paralysis after a minimum of 7 days and a maximum of 14 days after the onset of paralysis [3] to determine the prognosis of disease. However, the participants in our trial were those with sequelae of Bell’s palsy whose onsets were longer than six months. Moreover, stimulations such as inserting a needle electrode into the muscle or electrical stimulation during the electrodiagnostic test could influence the acupuncture treatment. Therefore, we did not assess the neurophysiological recovery of the facial nerves and muscles after acupuncture.

Collectively, the participants in our study complied well with the study protocols, with a dropout rate of less than 20 %.

## Conclusions

We conclude that acupuncture had a safe and partially beneficial effect on sequelae of Bell’s palsy, thereby demonstrating the feasibility of acupuncture as an effective means of treating sequelae of Bell’s palsy.

## Additional file

Additional file 1:
**CONSORT 2010 checklist of information to include when reporting a randomised trial*.**

## Abbreviations

ANCOVA: Analysis of covariance
CONSORT: Consolidating standards of reporting trials
FDI: Facial disability index
